# Human Papilloma Virus (HPV) prevalence and genotype distribution among women in Karachi, Pakistan

**DOI:** 10.1016/j.jve.2026.100631

**Published:** 2026-06-24

**Authors:** S. Maria Jilani, Angelika Iftner, Hana'a Iqbal, Atia Tul-Wahab, M. Iqbal Choudhary, Sharmeen Fayyaz, Tina M. Rehm, Thomas Iftner

**Affiliations:** a National Institute of Virology, Dr. Panjwani Center for Molecular Medicine and Drug Research, International Center for Chemical and Biological Sciences (ICCBS), University of Karachi, Karachi, 75270, Pakistan; b Dr. Panjwani Center for Molecular Medicine and Drug Research, International Center for Chemical and Biological Sciences (ICCBS), University of Karachi, Karachi, 75270, Pakistan; c Institute for Medical Virology and Epidemiology of Viral Diseases, University Hospital Tübingen, 72076, Tübingen, Germany

## Abstract

Human papillomavirus (HPV) epidemiological data from Pakistan remain limited, particularly in unscreened and unvaccinated populations. We performed a cross-sectional study in Karachi, Pakistan, to determine overall type- and age-specific HPV prevalence between May 2022 and November 2023. A total of 3119 women were invited to participate; 497 women gave consent, provided cervical samples and were included in the final analysis. HPV detection and genotyping were done using the highly sensitive INNO-LiPA HPV Genotyping Extra II line probe assay. Overall HPV prevalence was 16.7%. High-risk (HR) HPV types (counted once per genotype within IARC groups 1, 2A and 2B) were detected in 11.8% of women, and low-risk or unclassified types in 5.0%. Single HR-HPV infections occurred in 8.4% and multiple HR-HPV infections in 3.4%. Fourteen HR genotypes were detected as single infections, with HPV31 and HPV53 (1.2% each) being most prevalent, followed by HPV68 (1.0%), HPV16 (0.8%), and HPV33/HPV39/HPV51 (0.6% each). Less frequent types included HPV82 and HPV18 (0.4% each), and HPV35/HPV70/HPV73 (0.2% each). The most prevalent low-risk type was HPV6 (1.2%). HPV prevalence peaked in women aged 25-34 years (21.4%) and remained detectable in women above 54 years (12.5%). These data indicate a substantial and genetically diverse HR-HPV burden in Karachi. They support organized HPV-based cervical cancer screening using high-performance assays that detect the full panel of carcinogenic HPV types, with adequate triage and follow-up. They also support rapid scale-up and monitoring of HPV vaccination in Pakistan, including assessment of broader-valency vaccination strategies where feasible, to reduce cervical cancer, pre-cancerous lesions and other HPV-related cancers.

## 1 Introduction

Cervical cancer is the fourth most common cancer in women worldwide and the second most common cancer in women of reproductive age. With 662301 new cases and 348874 deaths worldwide in 2022, cervical cancer is a global health burden.^1^^,^^2^ It is particularly prevalent in developing countries, where about 85% of cervical cancer cases are diagnosed. In many developed countries, the incidence of cervical cancer has declined in recent decades following the introduction of organized cervical cancer screening programs using cervical cytology.^3^ In contrast, the incidence of cervical cancer remains high in developing countries because of a lack of organized screening and a high prevalence of risk factors for cervical neoplasia, particularly infection with high-risk human papillomavirus (HR-HPV) and co-infection with human immunodeficiency virus (HIV). Persistent infection with high risk HPV types (HR) has been identified as a necessary risk factor in the development of cervical cancer.^2^^,^^4^ Pakistan is the 5th most populous country in the world where the incidence of cancer is higher than in neighboring countries as there are no screening programs in place. According to the Globocan report, there were at least 4762 new cases of cervical cancer and 3069 deaths due to cervical cancer in Pakistan in 2022 and cervical cancer accounts for 2.3% of all cancer deaths among women in Pakistan.^5^

Human papillomaviruses (HPV) are small, double-stranded DNA viruses of the family Papillomaviridae that contain circular DNA of approximately 8000 base pairs and lack a lipid envelope. To date, more than 450 HPV types have been identified.^6^^,^^7^ Approximately 30 HPV types infect the genital mucosa, particularly in the anogenital tract of women and men. According to their carcinogenic potential, HPV are divided into High-risk and low-risk (LR) types. LR-types mainly cause warts in the external genital tract (e.g. condylomata acuminata) and low-grade cervical intraepithelial neoplasia (CIN1) at the cervix, whereas High-risk (HR) or potentially high-risk types are associated with the development of precursors and cervical, vulvar and anal cancers. HPV types 16, 18, 31, 33, 35, 39, 45, 51, 52, 56, 58, 59 and 68 are classified as group I/IIA human carcinogens or high risk (HR), while types 26, 53, 66, 67, 70, 73, 82 are classified as group IIB carcinogens^2^^,^^8^ or potential high risk. HPV types 6 and 11 are classified as low risk (LR) with lower evidence of carcinogenicity^9^ (Table 1). All other types affecting the anogenital tract have not yet been classified.^2^ A necessary risk factor for the development of cervical cancer is the persistent infection with HPV for more than 12 months. The persistence rate varies for different HPV types from 7% for HPV53 to 30% for HPV16.^10^ Cervical cytology-based screening programs were introduced by developed countries as early as 1970. Although this technique has been shown to reduce the incidence rate of cervical cancer, only few developing countries are using it. In the last decade cytology-based screening programs were replaced by co-testing or solely HPV-testing based primary screening programs. The incorporation of HPV molecular testing into cervical cancer screening programs enables earlier detection of relevant HPV types, which results in fewer cancer cases and fewer high-grade cervical intraepithelial neoplasia (CIN3) than with cytology alone 11, 12, 13, 14, 15, 16, 17, 18, 19. The lower risk following HPV testing suggests that extended screening intervals are appropriate,^16^^,^^17^^,^^20^ because this also avoids the detection of transient infections in successive screening rounds, which could lead to overtreatment.

**Table 1:** Classification of HPV-types according to IARC (^2^^,^^8^^,^^9^).

HPV type	Classification	
16	HR-HPV (G1)	High risk group 1
18	HR-HPV (G1)	
31	HR-HPV (G1)	
33	HR-HPV (G1)	
35	HR-HPV (G1)	
39	HR-HPV (G1)	
45	HR-HPV (G1)	
51	HR-HPV (G1)	
52	HR-HPV (G1)	
56	HR-HPV (G1)	
58	HR-HPV (G1)	
59	HR-HPV (G1)	
68	HR-HPV (G2A)	High risk group 2A
53	HR-HPV (G2B)	High risk group 2B
66	HR-HPV (G2B)	
67	HR-HPV (G2B)	
70	HR-HPV (G2B)	
73	HR-HPV (G2B)	
82	HR-HPV (G2B)	
6	LR-HPV	Low-risk
11	LR-HPV	
40	UC	unclassified
44	UC	
54	UC	
81	UC	
61	UC	
62	UC	
unknown type	HPVX	HPV type not included in type-specific detection

In Pakistan, the high prevalence of cervical cancer and associated morbidity is linked to the historical absence of organized HPV vaccination, lack of appropriate screening programs and late diagnosis. Pakistan launched its first large-scale HPV vaccination campaign for girls aged 9-14 years in 2025, after completion of the present sampling period, but adult screening-age women remain largely unvaccinated and require secondary prevention through screening.^21^ Women have little or no information about risk factors for cervical cancer, screening possibilities, HPV testing and cervical cytology has not been established. In addition, sexual norms in Pakistan differ from those in Western countries, and any discussion of sexuality and sexual behavior, including sexually transmitted infections, is generally considered a social taboo, minimizing awareness of HPV-related infections and HPV-associated cancers. Therefore, especially under these difficult conditions, it is crucial to conduct epidemiological studies to assess the underlying prevalence of HPV in Pakistani women to lay the foundation for future cervical cancer screening and vaccination programs.

Here we used the sensitive INNO Lipa Extra II line probe assay, based on the principle of reverse hybridization and designed to identify 32 different genotypes of human papillomavirus (HPV), to estimate the prevalence of HPV infection in women from the greater Karachi area of Pakistan with very high sensitivity. This highly sensitive INNO Lipa test has been demonstrated to identify the full spectrum of single and mixed genital HPV genotype infection. It was applied to get a broad picture of HPV infections present in the participating women, and is highly comparable to other PCR tests used in earlier studies in Pakistan. This was accompanied by a questionnaire to collect demographic and behavioral data on potential risk factors for HPV positivity. We found a rather high prevalence of HPV with up to 16.7% positive women aged 25-60 years seeking medical care for various reasons.

## 2 Material and methods

### 2.1 Study population and recruitment

This study was carried out in collaboration with the Gynecology departments of different hospitals. Recruitment of participants was carried out from May 2022 to November 2023. Hospitals were selected/contacted as to cover central, South, and west districts of Karachi and geographical clusters including Malir, Landhi, DHA, and HUB (at community level/municipalities), followed by recruitment of women aged 25 to 60 years in each of these clusters after giving consent.

### 2.2 Ethical approval

The independent ethics committee of International Center for Chemical and Biological Sciences (ICCBS) approved the study (Study# 076-PS-2022) and protocol as ICCBS/IEC-076- PS-2022/Protocol/1.0.

### 2.3 Sample collection

Cervical smear samples were collected from women who visited an outpatient gynecology department. In total 9 hospitals with gynecology departments situated in Karachi were contacted, and asked to participate in the study. Of these, 4 gynecologists from 5 hospitals agreed to participate in the study. The participants were briefed about the aims and objectives of the research and informed consent from women was obtained before inclusion in the study and sample collection. Women were excluded when pregnant, when they were under 25 or above 60, had amenorrhea, had undergone hysterectomy or active HIV or other immunosuppressive diseases. Participants or designated doctors were asked to fill out a questionnaire (Supl. Fig.1). The questionnaire was designed to accommodate different aspects: (1) general demographic information, (2) information regarding cancer screening and history, (3) information on sexual behavior (number of marriages), smoking habits and (4) information on previous knowledge or awareness about HPV screening. Invited participants were excluded from the study if they did not sign the consent form, or if no samples were provided. Only one woman gave consent, but could not be sampled because of above exclusion criteria.

### 2.4 HPV testing

Cervical smears were collected at the participating hospitals (Al-Riyaz hospital, Green star clinics, Medicell and concept fertility, New beginnings maternity care and Patel hospital) and transported to the National Institute of Virology at the ICCBS of the University of Karachi (NIV) at ambient temperature in viral transfer medium (Sansure Biotech, China). In total 497 samples were collected, frozen in transfer medium, and sent to the Institute for Medical Virology and Epidemiology of Viral Diseases, University Hospital Tuebingen, Germany. Samples were stored at −20 °C until processed.

### 2.5 DNA extraction

DNA was extracted using QIA amp DNA Mini Kit (#51306) and the QiaCube extraction system according to the manufacturer instructions (Qiagen, Germany). Up to 12 DNA-extractions per run fit into the machine, while a total volume of 200 μl of the sample was inserted per sample into the QiaCube for extraction. The. Purified DNA was eluted in 100 μl elution buffer and stored at −20 °C until further use.

### 2.6 HPV testing and genotyping

We used the INNO-LiPA HPV Genotyping Extra II assay (Fujirebio) to test for presence of HPV DNA. Testing was done according to the manufacturer's instructions. For this a LIPA-PCR was done with 10 μl of the purified DNA-solution and 40 μl of the master-mix with SPF10 specific primers. Per run, 46 patient samples are tested. A kit-internal positive control (HPV6) and a negative control (water) were processed in parallel for each run. The INNO-LiPA identifies 32 HPV genotypes of which 13 are defined as high-risk (HR) HPV types (HPV16/18/31/33/35/39/45/51/52/56/58/59/68), and the rest (6/11/26/40/42/43/44/53/54/61/62/67/66/70/73/81/82/83/89) were classified as low-risk, potentially high risk or unclassified types. Samples testing positive for HPV but without identifying a specific genotype were classified as HPV X (HPV control 1 or 2 positive). The test-strip includes four test-control lines. The test-strips were analyzed with an Epson Perfection V600 scanner and the specific software from Fujirebio (LiRAS for LiPA HPV-software). The scanner is calibrated once a month with calibration strips from the manufacturer to ensure valid quantification. Samples were declared positive, if all internal controls were valid (conjugation-control, human-DNA-control), and either one or multiple (1-7 of 12 possible) HPV bands were detected. In addition to the scanner, results were checked manually by naked eye. As external quality control for the process. Ring trials with other hospitals are done two times a year to ensure reproducibility and the laboratory is certified according to ISO 15189 Results were communicated to concerned gynecologist who are responsible for forwarding them to the participants.

## 3 Results

**Fig. 1:**
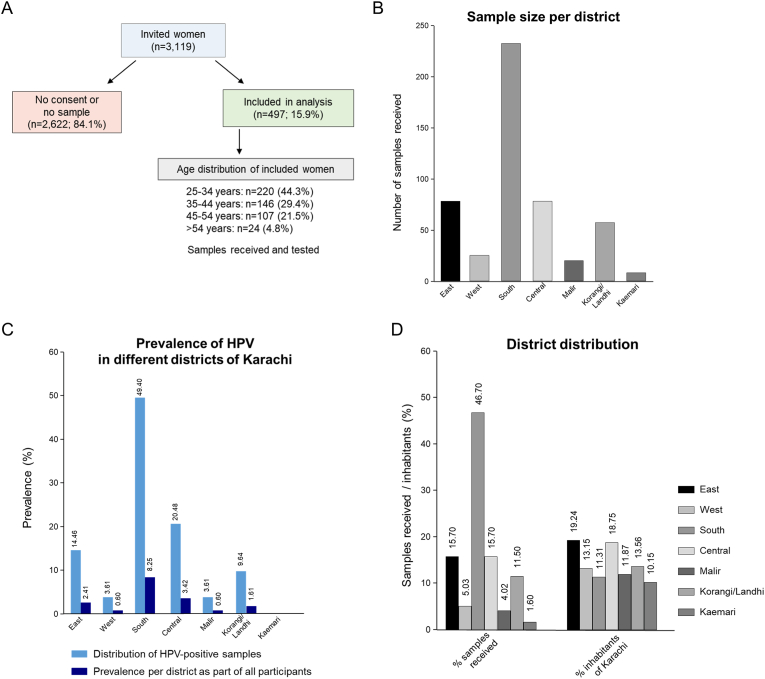
(A) Study cohort: number of invited women, women included in the final analysis and age distribution of tested participants. (B) Number of participants grouped by district of residence in Karachi. (C) Distribution of HPV-positive samples by district and prevalence per district as part of all participants. (D) District distribution in percent for tested participants and, for comparison, distribution of total inhabitants in Karachi in percent per district. District population percentages in panel D are based on 2023 census district data.^22^^,^^23^

In total, 3119 women were invited to participate in the study; 497 women provided consent and cervical samples and were included in the final analysis (Fig. 1A). Sampling could not be performed for all women who initially gave consent because of stenosis, fear of the sampling procedure or other practical constraints. Characteristics of the analyzed cohort are shown in Table 2.

**Table 2:** Characteristics of study participants divided into total, HPV-negative and HPV-positive participants.

Characteristic	Total participants:497	HPV-negative:414	HPV-positive:83
	n/497 (% of total)	n/414 (% of negative)	n/83 (% of positive)
**Region/district of Karachi**			
East	78 (15.69)	66 (15.94)	12 (14.46)
West (Orangi)	25 (5.03)	22 (5.31)	3 (3.61)
South	232 (46.68)	191 (46.14)	41 (49.4)
Central	78 (15.69)	61 (14.73)	17 (20.48)
Malir	20 (4.02)	17 (4.11)	03 (3.61)
Korangi/Landhi	57 (11.47)	49 (11.84)	08 (9.64)
Kaemari	08 (1.61)	08 (1.93)	0
**Smoking behaviour**			
No	398 (80.08)	334 (80.68)	64 (77.11)
Yes	99 (19.92)	80 (19.32)	19 (22.89)
**Awareness of PAP-test**			
Yes	54 (10.87)	46 (11.11)	08 (9.64)
No	443 (89.13)	368 (88.89)	75 (90.36)

### 3.1 Demographic characteristics of participants

Age ranged from 25 to 60 years, with a mean age of 37 years. Participants were grouped as 25-34 years (n = 220, 44.3%), 35-44 years (n = 146, 29.4%), 45-54 years (n = 107, 21.5%) and >54 years (n = 24, 4.8%) (Table 2; Fig. 1A). Most participants reported one marriage; only two participants reported more than one partner. Participants were recruited from different districts of Karachi, with 10 participants (2.04%) from Quetta, Baluchistan. District-level recruitment was uneven: the largest share of participants came from district South, followed by East, Central, Korangi/Landhi, West, Malir and Kaemari (Table 2; Fig. 1B–D). Because recruitment numbers differed between districts, district-level HPV percentages were interpreted descriptively. District South contributed the largest proportion of HPV-positive samples, which was consistent with the high proportion of participants recruited from that district (Fig. 1C).

### 3.2 Prevalence of HPV infections

**Fig. 2:**
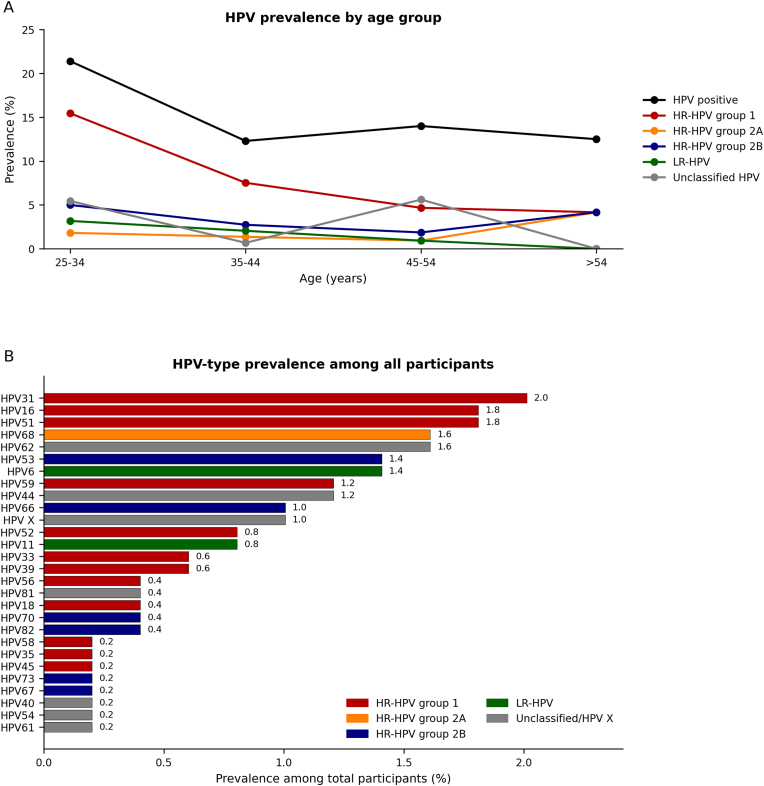
Prevalence of HPV infections. High-risk HPV (HR-HPV) group 1 is shown in red (carcinogenic), group 2A in orange (probably carcinogenic), group 2B in blue (potentially carcinogenic), low-risk HPV (LR-HPV) in green and unclassified/HPV X in grey, according to IARC classification. (A) Prevalence of HPV groups across age groups, including single and mixed infections. (B) Total type-specific prevalence of single and mixed infections, counted per genotype and ordered by prevalence.

Total HPV positivity among all age groups was 16.7% (83/497). High-risk HPV types (counted once per genotype within IARC Groups 1, 2A and 2B) were detected in 11.8% of women, whereas low-risk and unclassified types were detected in 5.0% (Table 3; Fig. 2A). Fourteen HR-HPV types were detected as single infections; HPV31 and HPV53 were most frequent (1.2% each), followed by HPV68 (1.0%), HPV16 (0.8%), and HPV33, HPV39 and HPV51 (0.6% each) (Table 4). The most prevalent low-risk type detected as a single infection was HPV6 (1.2%). The highest HPV prevalence was observed in women aged 25-34 years (47/220; 21.4%); in women >54 years, HPV was detected in 3/24 (12.5%) (Table 3; Fig. 2A). Single HPV infections were observed in 63/497 (12.7%), double infections in 14/497 (2.8%) and multiple infections in 6/497 (1.2%) (Table 3). Single HR-HPV infections were observed in 8.4%, while multiple HR-HPV infections were present in 3.4% of the women tested. When single and multiple infections were combined, HPV31 (10/497; 2.0%), HPV16 and HPV51 (9/497; 1.8% each) were the most frequent HR-HPV genotypes (Table 5; Fig. 2B). HPV45, HPV52, HPV54, HPV58, HPV59 and HPV67 were observed only in multiple infections, whereas HPV26, HPV30, HPV34, HPV69 and HPV80 were not detected.

**Table 3:** HPV prevalence within age groups and distribution of single, double and multiple HPV infections.

	Age groups				total
Age (years):	25-34	35-44	45-54	>54	
**HPV-negative n (frequency of total in %):**	173/497 (34.8)	128/497 (25.8)	92/497 (18.5)	21/497 (4.2)	414/497 (83.3)
**Sample size n:**	220	146	107	24	497
**HPV-positive n (frequency in %):**	47/220 (21.4)	18/146 (12.3)	15/107 (14.0)	3/24 (12.5)	83/497 (16.7)
**Single HPV: (including HPV X)**	32/220 (14.54)	16/146 (10.95)	12/107 (11.21)	3/24 (12.5)	63 (12.67)
**Double HPV:**	10/220 (4.54)	1/146 (0.68)	3/107 (2.80)	0	14 (2.81)
**Multiple HPV:**	5/220 (2.27)	1/146 (0.68)	0	0	6 (1.20)

**Table 4:** Prevalence of single infections of high- and low-risk, and unclassified and unidentified HPV types in age groups, ordered by classification in descending order of total prevalence per type.

Age group (years):	25-34	35-44	45-54	>54	Total
Number of participants:	220	146	107	24	497
Number of positive participants:	n/220 (%)	n/146 (%)	n/107 (%)	n/24 (%)	n/497 (%)
**HR-HPV (G1)**					
**31**	2 (0.9)	2 (1.37)	2 (1.87)	1	6 (1.21)
**16**	3 (1.36)	1 (0.68)	0	0	4 (0.80)
**51**	1 (0.45)	2 (1.37)	0	0	3 (0.60)
**59**	0	0	0	0	0
**52**	0	0	0	0	0
**33**	0	1 (0.68)	1 (0.93)	1 (4.17)	3 (0.60)
**39**	2 (0.9)	1 (0.68)	0	0	3 (0.60)
**18**	2 (0.9)	0	0	0	2 (0.40)
**56**	1 (0.45)	0	0	0	1 (0.20)
**35**	1 (0.45)	0	0	0	1 (0.20)
**45**	0	0	0	0	0
**58**	0	0	0	0	0
**HR-HPV (G2A)**					
**68**	1 (0.45)	2 (1.37)	1 (0.93)	1 (4.17)	5 (1.01)
**HR-HPV (G2B)**					
**53**	3 (1.36)	2 (1.37)	1 (0.93)	0	6 (1.21)
**66**	2 (0.9)	0	1 (0.93)	1 (4.17)	4 (0.80)
**70**	1 (0.45)	0	0	0	1 (0.20)
**82**	2 (0.9)	0	0	0	2 (0.40)
**73**	0	1 (0.68)	0	0	1 (0.20)
**67**	0	0	0	0	0
**LR-HPV**					
**6**	3 (1.36)	3 (2.05)	0	0	6 (1.21)
**11**	1 (0.45)	0	0	0	1 (0.20)
**Unclassified HPV**					
**62**	3 (1.36)	1 (0.68)	1 (0.93)	0	5 (1.01)
**44**	0	0	1 (0.93)	0	1 (0.20)
**81**	1 (0.45)	0	0	0	1 (0.20)
**40**	1 (0.45)	0	0	0	1 (0.20)
**54**	0	0	0	0	0
**61**	0	0	1 (0.93)	0	1 (0.20)
**HPV X (not identified)**					
**-**	1 (0.45)	1 (0.68)	3 (2.80)	0	5 (1.01)

**Table 5:** Total prevalence (single and multiple infections summed up counted per type) of high- and low-risk, and unclassified and unidentified HPV types in age groups, ordered by classification in descending order of total prevalence per type.

Age group (yaers):	25-34	35-44	45-54	>54	Total
number of participants	220	146	107	24	497
sum per HPV-type	n/220 (%)	n/146 (%)	n/107 (%)	n/24 (%)	n/497 (%)
**HR-HPV (G1)**					
**31**	5 (2.27)	2 (1.37)	3 (2.80)	0	10 (2.01)
**16**	8 (3.6)	1 (0.68)	0	0	9 (1.81)
**51**	4 (1.8)	4 (2.74)	1 (0.93)	0	9 (1.81)
**59**	5 (2.27)	1 (0.68)	0	0	6 (1.21)
**52**	3 (1.36)	1 (0.68)	0	0	4 (0.80)
**33**	0	1 (0.68)	1 (0.93)	1 (4.17)	3 (0.60)
**39**	2 (0.90)	1 (0.68)	0	0	3 (0.60)
**18**	2 (0.90)	0	0	0	2 (0.40)
**56**	2 (0.9)	0	0	0	2 (0.40)
**35**	1 (0.45)	0	0	0	1 (0.20)
**45**	1 (0.45)	0	0	0	1 (0.20)
**58**	1 (0.45)	0	0	0	1 (0.20)
**HR-HPV (G2A)**					
**68**	4 (1.8)	2 (1.37)	1 (0.93)	1 (4.17)	8 (1.61)
**HR-HPV (G2B)**					
**53**	4 (1.8)	2 (1.37)	1 (0.93)	0	7 (1.41)
**66**	2 (0.90)	1 (0.68)	1 (0.93)	1 (4.17)	5 (1.01)
**70**	2 (0.90)	0	0	0	2 (0.40)
**82**	2 (0.90)	0	0	0	2 (0.40)
**73**	0	1 (0.68)	0	0	1 (0.20)
**67**	1 (0.45)	0	0	0	1 (0.20)
**LR-HPV**					
**6**	4 (1.8)	3 (2.05)	0	0	7 (1.41)
**11**	3 (1.36)	0	1 (0.93)	0	4 (0.80)
**Unclassified HPV**					
**62**	5 (2.27)	1 (0.68)	2 (1.87)	0	8 (1.61)
**44**	3 (1.36)	0	3 (2.80)	0	6 (1.21)
**81**	2 (0.90)	0	0	0	2 (0.40)
**40**	1 (0.45)	0	0	0	1 (0.20)
**54**	1 (0.45)	0	0	0	1 (0.20)
**61**	0	0	1 (0.93)	0	1 (0.20)
**HPV X (not identified)**					
**-**	2 (0.90)	0	3 (2.80)	0	5 (1.01)

### 3.3 HPV genotype distribution in women across different age groups

Prevalence of HPV infections in different age groups is presented in Table 3, Table 4, Table 5. Women were classified into four age groups: 25-34 years, 35-44 years, 45-54 years and >54 years. LR-HPV detection decreased with increasing age, from 3.2% (7/220) in women aged 25-34 years to 2.1% (3/146), 0.9% (1/107) and 0% in the older age groups (Table 5; Fig. 2A). When single and multiple infections were counted together, HPV16 was detected in 8/220 (3.6%) and HPV31 in 5/220 (2.3%) of women aged 25-34 years; HPV51, HPV68 and HPV53 were each detected in 4/220 (1.8%) in this age group (Table 5).

### 3.4 Risk factors for HPV infection

Smoking was reported by 99/497 (19.9%) participants. HPV positivity did not differ significantly by smoking status (Fisher's exact test: p = 0.45; odds ratio 1.24; 95% confidence interval 0.70-2.20; Table 2). Two women reported more than one partner, and both were HR-HPV-positive; because of the small number, further statistical analysis was not performed. Overall, 352/497 (70.8%) participants reported at least one gynecological symptom, whereas 145/497 (29.2%) did not (Table 2). HPV positivity did not differ significantly by symptom status (Fisher's exact test: p = 0.60; odds ratio 1.17; 95% confidence interval 0.70-1.99).

## 4 Discussion

Cervical cancer remains a major public-health challenge in Pakistan and other low- and middle-income countries, where cancer prevention is limited by low screening coverage, late presentation and restricted access to treatment.^1^^,^^3^^,^^5^^,^^24^^,^^25^^,^^26^ In the present clinic-based cohort from Karachi, we found an overall HPV prevalence of 16.7% and an HR-HPV prevalence of 11.8% using the highly sensitive INNO-LiPA HPV Genotyping Extra II assay. This indicates a substantial burden of cervical HPV infection among adult women seeking gynecological care in Karachi. Because the cohort was not population-based and because the assay can detect low-copy, transient or latent infections, this estimate should not be interpreted as a national population prevalence. Nevertheless, it provides important local genotype data from a setting where organized HPV-based screening has not yet been implemented.

The prevalence observed in this study is higher than the HPV prevalence reported in earlier studies from Pakistan, in which overall HPV positivity ranged from approximately 2.8% to 4.7% in women without cervical cancer or in screening cohorts.^27^^,^^28^ Several factors may explain the difference, including differences in recruitment, age structure, regional population composition, sample collection and the analytical sensitivity of the HPV detection methods. The INNO-LiPA assay was intentionally selected to capture a broad spectrum of single and mixed HPV infections; therefore, it is expected to detect more infections than less sensitive assays or assays targeting fewer genotypes.^29^ The finding that HPV positivity peaked in women aged 25-34 years and declined thereafter is consistent with the well-described age pattern of HPV acquisition and clearance after sexual debut.^30^^,^^31^ The continued detection of HPV in women >54 years in our cohort indicates that screening should not be restricted to young women only, especially in a population without previous organized screening.

The genotype distribution in Karachi differed from the pattern typically expected from invasive cervical cancer series. In our cohort, HPV31, HPV16, HPV51, HPV68, HPV53 and HPV59 were among the most frequently detected HR or potentially HR genotypes when single and multiple infections were counted together, whereas HPV18 and HPV58 were uncommon. This does not contradict the central role of HPV16 and HPV18 in cervical carcinogenesis, because genotype distribution in prevalent cervical infections differs from genotype attribution in invasive cancer. In Pakistan, HPV16/18 are estimated to account for a large proportion of invasive cervical cancers,^26^ while our data describe prevalent infections in mostly screening-age women without histological classification. The relatively high representation of non-16/18 HR types therefore supports the need for screening assays that detect the full panel of clinically relevant HR-HPV genotypes.

Comparison with India is particularly informative because both countries are in South Asia but differ substantially in health-system organization, screening coverage and available HPV data. Recent Indian estimates suggest a pooled HR-HPV prevalence of 11.4% in the general female population and a pooled HPV16/18 prevalence of 5.6%.^32^ The HR-HPV prevalence in our Karachi cohort (11.8%) was therefore close to the pooled Indian HR-HPV estimate, but the type distribution differed. Indian summary data for women with normal cytology show HPV16 and HPV18 as the most prominent vaccine-targeted types, with HPV16 at 3.6% and HPV18 at 1.4%, while HPV31, HPV33, HPV35, HPV52 and HPV58 occur at lower type-specific prevalence.^33^ In contrast, our study found HPV31 at the top of the single infection genotype distribution, HPV16 at lower prevalence than in many Indian datasets, and very low HPV18 prevalence. These differences suggest that extrapolation from Indian HPV genotype data to Pakistan may be misleading and that Pakistan needs its own regional HPV surveillance to guide screening algorithms, vaccine-impact monitoring and future vaccine-policy decisions.

The clinical implication for cervical cancer screening in Pakistan is that HPV-based screening should not be limited to HPV16/18 genotyping alone. A strategy that tests only for HPV16 and HPV18 would miss a large proportion of HR-HPV infections detected in this study. For primary screening, Pakistan should therefore prioritize clinically validated high-performance HPV tests that detect all carcinogenic HPV types, followed by an appropriate triage pathway such as partial genotyping, visual assessment, or other locally feasible triage tools. This approach is aligned with WHO elimination targets, which call for 70% of women to be screened with a high-performance test by ages 35 and 45 years, together with 90% treatment coverage for women with detected cervical disease.^34^ In our cohort, only 10.9% of women reported awareness of Pap testing, which underscores the need for culturally sensitive education, provider training, self-sampling options and referral systems that can function in routine Pakistani health-care settings.^35^

The implications for vaccination are complementary but not identical to those for screening. Pakistan's first large-scale HPV vaccination campaign, launched in 2025, is an important step toward primary prevention and aims to reach girls aged 9-14 years before HPV exposure.^21^ Even though HPV16/18 were not the dominant prevalent genotypes as single infections, HPV16 was found the most prevalent type in multiple infections, so vaccination against HPV16/18 remains essential. However, our finding of diverse non-16/18 HR-HPV infections indicates that vaccine impact should be monitored by genotype-specific surveillance and that broader-valency vaccines, where affordable and programmatically feasible, could provide additional prevention against types such as HPV31, HPV33, HPV45, HPV52 and HPV58, as well as HPV6/11-associated genital warts. Because current vaccines do not cover all genotypes, vaccination cannot replace screening for adult women and should be implemented as part of an integrated prevention program.

This study has limitations. The samples were clinic-based and participants were recruited in selected hospitals and districts of Karachi, so it may not represent the general female population of Karachi or Pakistan. District-level estimates should be interpreted descriptively because the recruitment numbers differed substantially between districts. We did not include cytology or histology as disease endpoints, as they were not available, and the cross-sectional design cannot distinguish transient from persistent infection. Sexual behavior and other risk-factor variables may also be underreported because of cultural sensitivities around these topics. Despite these limitations, the study provides a broad, sensitive assessment of HPV genotype diversity in an underserved and unvaccinated adult female population in Pakistan.

In conclusion, our data shows a substantial and heterogeneous HR-HPV burden among women attending gynecological care in Karachi. The HR-HPV prevalence was comparable to recent estimates from India, but the genotype distribution differed, with a notable contribution from HPV31 and other non-16/18 HR types. For Pakistan, these findings support the urgent implementation of organized HPV-based screening using assays with broad HR-HPV genotype coverage, clear triage and follow-up pathways, and strong educational components. In parallel, high-coverage HPV vaccination of adolescents should be scaled up and monitored by genotype-specific surveillance. Together, these measures could reduce future cervical cancer incidence and mortality while generating the evidence base needed for locally adapted cervical cancer elimination strategies in Pakistan.

## CRediT authorship contribution statement

S. Maria Jilani: Sample collection, Methodology, Investigation. Angelika Iftner: Methodology, formal analysis. Hana'a Iqbal: review & editing. Atia Tul-Wahab: Resources. M. Iqbal Choudhary: Supervision, Resources, Funding acquisition. Sharmeen Fayyaz: Writing – review & editing, Supervision. Tina M. Rehm: Writing – review & editing. Thomas Iftner: Writing – original draft, Supervision, Resources, Project administration, Investigation, Conceptualization, Formal analysis.

## Declaration of competing interest

None.

## Data Availability

Data will be made available on request.

## Appendix A Supplementary data

Multimedia component 1 Empty questionnaire and informed consent form used in our study.

## References

[1] Li Z. (2025). Global landscape of cervical cancer incidence and mortality in 2022 and predictions to 2030: the urgent need to address inequalities in cervical cancer. Int J Cancer.

[2] IARC, A review of human carcinogens (2012).

[3] Vaccarella S. (2014). 50 years of screening in the nordic countries: quantifying the effects on cervical cancer incidence. Br J Cancer.

[4] Walboomers J.M. (1999). Human papillomavirus is a necessary cause of invasive cervical cancer worldwide. J Pathol.

[5] Ferlay J., Ervik M., Lam F. (2024). https://gco.iarc.who.int/today.

[6] National Institute of Allergy and Infectious Diseases PaVE: Papillomavirus Episteme. https://pave.niaid.nih.gov/.

[7] Van Doorslaer K. (2017). The papillomavirus episteme: a major update to the papillomavirus sequence database. Nucleic Acids Res.

[8] Wei F. (2024). Causal attribution of human papillomavirus genotypes to invasive cervical cancer worldwide: a systematic analysis of the global literature. Lancet.

[9] IARC, Human papillomaviruses. Volume 90 (2007).

[10] Nielsen A. (2010). Persistence of high-risk human papillomavirus infection in a population-based cohort of Danish women. J Med Virol.

[11] Kitchener H.C. (2009). HPV testing in combination with liquid-based cytology in primary cervical screening (ARTISTIC): a randomised controlled trial. Lancet Oncol.

[12] Ronco G. (2010). Efficacy of human papillomavirus testing for the detection of invasive cervical cancers and cervical intraepithelial neoplasia: a randomised controlled trial. Lancet Oncol.

[13] Ronco G. (2006). Human papillomavirus testing and liquid-based cytology in primary screening of women younger than 35 years: results at recruitment for a randomised controlled trial. Lancet Oncol.

[14] Castle P.E. (2011). Performance of carcinogenic human papillomavirus (HPV) testing and HPV16 or HPV18 genotyping for cervical cancer screening of women aged 25 years and older: a subanalysis of the ATHENA study. Lancet Oncol.

[15] Stoler M.H. (2011). High-risk human papillomavirus testing in women with ASC-US cytology: results from the ATHENA HPV study. Am J Clin Pathol.

[16] Arbyn M. (2012). Evidence regarding human papillomavirus testing in secondary prevention of cervical cancer. Vaccine.

[17] Ronco G. (2014). Efficacy of HPV-based screening for prevention of invasive cervical cancer: follow-up of four European randomised controlled trials. Lancet.

[18] Bulkmans N.W. (2007). Human papillomavirus DNA testing for the detection of cervical intraepithelial neoplasia grade 3 and cancer: 5-year follow-up of a randomised controlled implementation trial. Lancet.

[19] Naucler P. (2007). Human papillomavirus and Papanicolaou tests to screen for cervical cancer. N Engl J Med.

[20] Sasieni P., Cuzick J. (2002). Could HPV testing become the sole primary cervical screening test?. J Med Screen.

[21] World Health Organization Regional Office for the Eastern Mediterranean (2025). Pakistan joins 150 countries to protect 13 million girls from cervical cancer with WHO-prequalified vaccine. https://www.emro.who.int/pak/pakistan-news/pakistan-joins-150-countries-to-protect-13-million-girls-from-cervical-cancer-with-who-prequalified-vaccine.html.

[22] Pakistan Bureau of Statistics (2024). https://www.pbs.gov.pk/wp-content/uploads/2020/07/National-Census-Report-2023.pdf.

[23] Pakistan Bureau of Statistics (2026). https://psi.pbos.gov.pk/.

[24] Batool S.A., Sajjad S., Malik H. (2017). Cervical cancer in Pakistan: a review. J Pakistan Med Assoc.

[25] Chughtai N. (2023). National cervical cancer burden estimation through systematic review and analysis of publicly available data in Pakistan. BMC Public Health.

[26] ICO/IARC Information Centre on HPV and Cancer (2023). https://hpvcentre.net/statistics/reports/PAK_FS.pdf.

[27] Raza S.A. (2010). Human papillomavirus infection in women with and without cervical cancer in Karachi, Pakistan. Br J Cancer.

[28] Shahid M. (2015). Cervical cancer screening and HPV genotype distribution among asymptomatic patients of Karachi Pakistan. Pakistan J Med Sci.

[29] Hebnes J.B. (2015). Human papillomavirus infection among 2460 men in Denmark: prevalence in relation to age using 2 human papillomavirus DNA testing methods. Sex Transm Dis.

[30] Castellsague X. (2012). Prevalence and genotype distribution of human papillomavirus infection of the cervix in Spain: the CLEOPATRE study. J Med Virol.

[31] de Sanjose S. (2007). Worldwide prevalence and genotype distribution of cervical human papillomavirus DNA in women with normal cytology: a meta-analysis. Lancet Infect Dis.

[32] Adhikari I., Kataria I., Bhandari P. (2026). Systematic review and meta-analysis of cervico-vaginal high-risk human papillomavirus prevalence in India prior to nationwide human papillomavirus vaccination. Arch Public Health.

[33] ICO/IARC Information Centre on HPV and Cancer. India (2023). https://hpvcentre.net/statistics/reports/IND.pdf.

[34] World Health Organization (2020). https://www.who.int/publications/i/item/9789240014107.

[35] Khan F.Z.A., Mazhar S.B., Itua I. (2025). Knowledge, attitudes and practices of cervical cancer screening among women attending gynecology clinics at tertiary care hospitals in the capital city of Pakistan: a cross-sectional survey. Asian Pac J Cancer Prev APJCP.

