# Supplementary material for: Human Papilloma Virus (HPV) prevalence and genotype distribution among women in Karachi, Pakistan
Source: J Virus Erad. 2026 Jun 24;12(3):100631. doi: 10.1016/j.jve.2026.100631 (PMC13331975; doi:10.1016/j.jve.2026.100631)
Supplement: Multimedia component 1 — Empty questionnaire and informed consent form used in our study. [file mmc1.pdf]

QUESTIONNAIRE

1. Patient information

Patient ID #: _________________
Patient first name: ________________
Age: _______________
Parity: ________________
Occupation: _______________________
Cell no: ______________________
Residence:
____
____
____

2. Questions

1. Marital status?
   - Unmarried
   - Married
   - If married, is it your first marriage?

   ____

2. Have you ever heard of a Pap smear (also called a Pap test)?
   - YES
   - NO

3. Have you had a Pap smear in the last 5 years?
   - Yes, reason

     ____
     ____
     ____

   - No, please indicate reasons and select all that apply:
   - Not aware of the test
   - Not convenient/no time
   - Fear
   - No symptoms, so test is not needed
   - Other, please specify________

4. Do you smoke?
   - Yes
   - No

5. Have you had any contraceptive?
    - IUD
    - Birth control pills
    - No, I don't have

6. Have you ever had any other sexually transmitted disease?
    - Syphilis
    - Chlamydia
    - If other than specify ,______________________________

7. Have you ever had any of these symptoms?
    - Discomfort in vagina
    - Irritation in vagina (tickling sensation)
    - Unusual vaginal discharge
    - Warts formation on cervix

8. Have you ever been tested for HPV (human papilloma virus)?
    - Yes
    - No
9. If yes, then what was the result?
    - Positive
    - Negative
10. If positive, then did you had treatment?
    - Yes
    - No

Data Collector Information:
Name: ______________
Institute: _____________________
Contact no: _________________________
Email: _________________________

Signature of data collector:

INFORMED CONSENT FORM

The informed consent form consists of two parts:

- Information sheet (to share information about the research)
- Certificate of consent (for signatures if agree to take part)

PART I: INFORMATION SHEET

Purpose of study:

To investigate the prevalence of human papilloma virus in general female population.

To investigate the type of human papilloma virus present in the women based population.

Methodology:

- Pap smears will be collected after the consent.
- Detection will be performed at NIV, ICCBS.
- Furthermore, genotyping will be done.

Risk from the study:

There is no potential risk to the patient after the sampling.

Benefit from the study:

The potential benefits include:

- Screening of HPV in general population
- Generation of epidemiological data of HPV
- Generation of data presenting type prevalent in Karachi based women population.
- Prevention of HPV related cancers if screened and treated beforehand.
- Urging this screening to be routine procedure in every hospital.

Confidentiality:

The information that we collect from this research project will be kept as confidential.it will not be shared with or given to anyone.

PATIENT CONSENT

I ___________________________ d/o ________________________ aged _________

under the treatment of Dr. ________________ do hereby give consent to the sampling.

The nature and purpose has been explained to me by the doctor.

I declare that I am more than 18 years of age.

I have been informed that these samples will be given for the research purpose, and I have no issue regarding this.

I have given this consent voluntarily out of my free will without any pressure.

Place:

Date and time:

Signature of patient:
